# ZnS, CdS and HgS Nanoparticles via Alkyl-Phenyl Dithiocarbamate Complexes as Single Source Precursors

**DOI:** 10.3390/ijms12095538

**Published:** 2011-08-29

**Authors:** Damian C. Onwudiwe, Peter A. Ajibade

**Affiliations:** Department of Chemistry, University of Fort Hare, Private Bag X1314, Alice 5700, South Africa; E-Mail: donwudiwe@ufh.ac.za

**Keywords:** metal sulfide, dithiocarbamates, nanoparticles, single source precursor

## Abstract

The synthesis of II-VI semiconductor nanoparticles obtained by the thermolysis of certain group 12 metal complexes as precursors is reported. Thermogravimetric analysis of the single source precursors showed sharp decomposition leading to their respective metal sulfides. The structural and optical properties of the prepared nanoparticles were characterized by means of X-ray diffraction (XRD), transmission electron microscopy (TEM), scanning electron microscopy (SEM) UV-Vis and photoluminescence spectroscopy. The X-ray diffraction pattern showed that the prepared ZnS nanoparticles have a cubic sphalerite structure; the CdS indicates a hexagonal phase and the HgS show the presence of metacinnabar phase. The TEM image demonstrates that the ZnS nanoparticles are dot-shaped, the CdS and the HgS clearly showed a rice and spherical morphology respectively. The UV-Vis spectra exhibited a blue-shift with respect to that of the bulk samples which is attributed to the quantum size effect. The band gap of the samples have been calculated from absorption spectra and werefound to be about 4.33 eV (286 nm), 2.91 eV (426 nm) and 4.27 eV (290 nm) for the ZnS, CdS and HgS samples respectively.

## 1. Introduction

Nanocrystalline materials have attracted much attention in recent years because properties in nanoforms differ significantly from those of their bulk counterparts [1]. Many fundamental properties of materials (optical, electrical, mechanical, *etc*.) can be expressed as a function of their size, composition and structural order [2]. When the size of a semiconductor becomes comparable to the 1S-exciton diameter, they exhibit quantum confinement [3–6]. This results in the appearance of a quantized Eigen spectrum and an increase in the energy gap relative to the band gap (Eg) of the bulk solid [7]. Consequently, much effort has been made to control the size, morphology and crystallinity of nanocrystals with a view to tune their physical properties.

The group IIB–VI nanoparticles are important semiconductor compounds and one of the most explored because of their wide range of potential applications. ZnS has a wide direct band gap of 3.65 eV (bulk) [8] and it is a promising material for optoelectronic device applications such as optical coatings, solid-state solar cell windows, electrooptic modulators, photoconductors, field effect transistors, sensors, transductors, light-emitting applications, and photonic crystal devices which operate in the region from visible to near infrared [9–11]. CdS nanoparticles are the most studied system among all the II-VI semiconducting nanoparticles [12]. Bulk CdS has a direct band gap of 2.42 eV at 300 K and a typical Bohr exciton diameter of around 5.8 nm. As a result, in the size range of 1–6 nm, CdS nanoparticles show sizable quantum confinement effects [13]. Cadmium sulfide shows great potential for uses in photochemical catalysis, solar cells, and nonlinear optical materials [14], and could be used as bioorganic detector of proteins [15] or DNA [16,17]. Nanocrystalline HgS has pronounced dichorism [18], photoelectric [19], acousto-optic properties [20] and electrostatic image properties [21]. Thus, it is a well known technologically important material. Despite these applications of HgS nanoparticles, relatively scarce studies are available due to difficulty in synthesis and toxicity of mercury. Only few detailed studies are available on preparation and characterization of HgS nanoparticles [22–25].

The use of single source precursors for the synthesis of metal chalcogenide nanoparticles has proven to be efficient routes for the synthesis of high quality nanocrystals. Ligands properties of the metal complexes used as precursor could be used in the modification of the size and shape of the nanoparticles. For example, variation of the alkyl groups on the dithiocarbamate ligand was found to give particles with non-spherical morphologies [26]. In this paper, group 12 complexes of mixed alkyl-phenyl dithiocarbamate complexes have been used as single source precursors to prepare ZnS, CdS, and HgS semiconductor nanoparticles.

## 2. Results and Discussion

### 2.1. Synthesis

The synthesis and characterization of the precursor complexes [ML^1^L^2^] (where M = Zn, Cd, and Hg; L^1^ = N-ethyl-N-phenyl dithiocarbamate and L^2^ = *N*-butyl-*N*-phenyl dithiocarbamate) have been reported [27]. The thermolysis of the complexes was carried out at 180 °C and in the presence of hexadecylamine as the capping agent. The appearance of pale yellow coloration for the Cd(II) complex, milky-white for the Zn(II) complex and grayish-black for the Hg(II) complex samples indicated the formation of CdS, ZnS and HgS nanoparticles. The function of the hexadecylamine was to solve the problem of insufficient dispersion of nanoparticles and formation of large aggregates which could result into the loss of the special nanoscale properties. Different types of surface-capping agents have been used to stabilize II-VI semiconductor nanoparticles. These include starch [28], polyphosphate [29], trioctylphosphine/trioctylphosphine oxide, and thiols [30]; they are capable of tuning nanoparticle shape, size, and other surface properties to different extents depending on their molecular structure. They also play a significant role in the transfer of the photo-generated electrons and holes to capping agents [31,32] and this plays an important role in determining the luminescence properties of the nanoparticles.

### 2.2. Thermal Decomposition Studies

In order to evaluate the potentials of the complexes as single source precursors, thermal decomposition profile of the complexes were studied using thermogravimetric analyzer and differential scanning calorimeter (DSC), under nitrogen condition. The temperature ranges and percentage mass losses during the decompositions as well as the temperature corresponding to the maximum rate of decomposition, DTG_max_ and the theoretical percentage mass losses are presented in Table 1.

**Figure 1:**
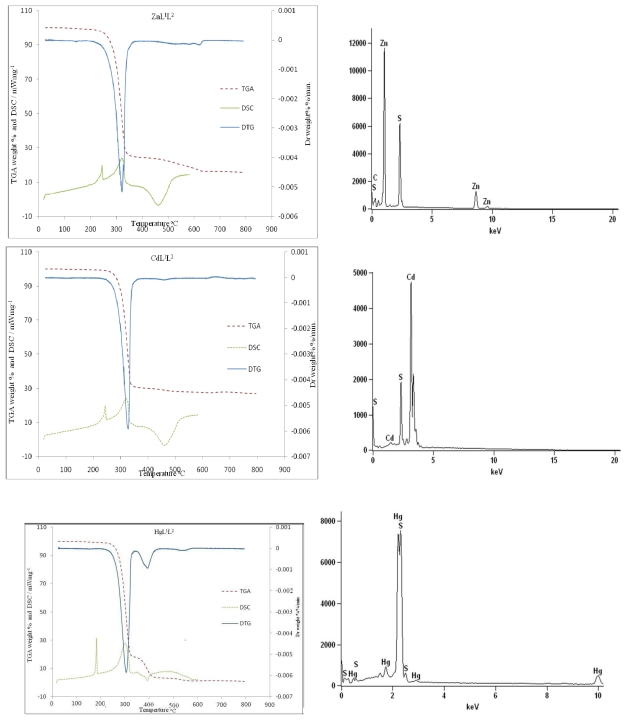
Thethermal gravimetric (TG), differential thermal gravimetric (DTG) and differential scanning calorimetric (DSC) curves of the complexes in nitrogen atmosphere and energy dispersive X-ray spectroscopic (EDX) analyses of the residue.

The thermograms of the complexes shown in Figures 1 for the Zn(II), Cd(II) and Hg(II) complexes respectively indicate that there is no appreciable weight loss up to 200 °C indicating the absence of small molecules of either water or solvent. The complexes decompose in two stages. The first stage, which is the major decomposition stage, is accompanied by a mass loss in the range 70–78% and it begins in the range 204–229 °C and terminates in the range 340–382 °C. The observed mass loss correspond to the breaking off of the ligand moiety given residues whose mass corresponds to the respective metal sulfides. The rate of mass loss reaches its maximum in the range 302–323 °C as is evident from the respective differential thermal gravimetric (DTG) peaks. This first decomposition stage is of importance to the synthesis of nanoparticles and to further ascertain the elemental composition of the products, the residues were analyzed using energy dispersive X-ray spectroscopic (EDX) analysis. The peaks observed were only the respective metals (Zn, Cd and Hg) and the sulfides (Figures 1); and confirms the suitability of the complexes as single source precursor complexes.

In the differential scanning calorimetric analysis, the first endothermic peak temperature in all the complexes is sharp and without a mass loss occurring in the range 181–260 °C is attributed to a melting event. In addition to these endothermic peak temperatures, there are exothermic peaks centered in the range 306–315 °C in all the complexes; a broad endothermic peak at 449 °C in the zinc complex, 451 °C in the cadmium complex, and 381 °C in the mercury complex. The endothermic peak temperatures in all the complexes correspond to the highest weight loss in the thermograms and are associated with decomposition or fragmentation of the organic moiety and subsequent formation of the respective metal sulfides. Above 400 °C, in the zinc and cadmium complexes, an exothermic broad peak is observed. These characteristic broad peaks have been observed in literature [33] and were ascribed to possible oxidation reaction.

### 2.3. Morphological Characterization

#### 2.3.1. Scanning Electron Microscopy (SEM)/Transmission Electron Microscopy (TEM) Studies

**Figure 2:**
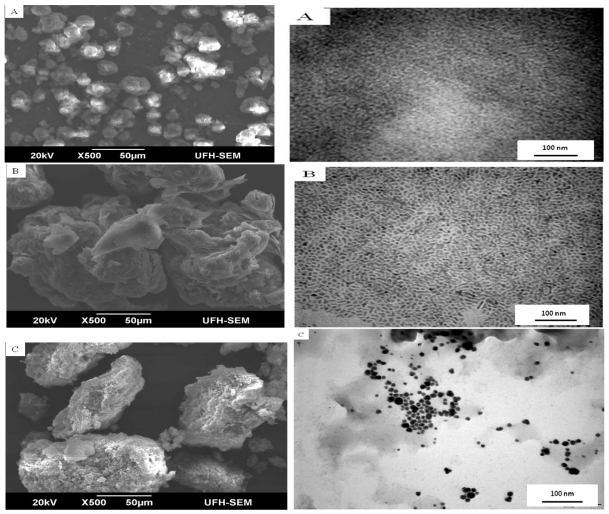
SEM/TEM images of ZnS (**A**), CdS (**B**) and HgS (**C**) nanoparticles.

The morphology and size of the as prepared metal sulfide samples are determined by SEM analysis. Figures 2 show the SEM micrographs of above prepared samples. The as prepared ZnS samples are spherical. The shape of the particles is regular with average size of approximately 15 μm. The uniformity and similar dimension of spheres show a good growth environment of ZnS crystals, which indicate good crystallinity of the spheres from the other aspect [34]. The CdS and HgS nanoparticles are spherical and agglomerated to form larger particles (Figure 2), and this makes the estimation of size difficult.

The TEM images (Figure 2) of the metal sulfide nanocrystals. The nanoparticles showed different shapes and sizes. The ZnS displayed dot-shaped particles; CdS showed close-to rice shaped structure while the HgS nanoparticles, under the same synthetic conditions, produced spherical and oval shapes with a good uniform size distribution throughout the sample. The sizes range from 10 to 30 nm (Figure 2).

#### 2.3.2. X-ray Diffraction (XRD) Patterns

**Figure 3:**
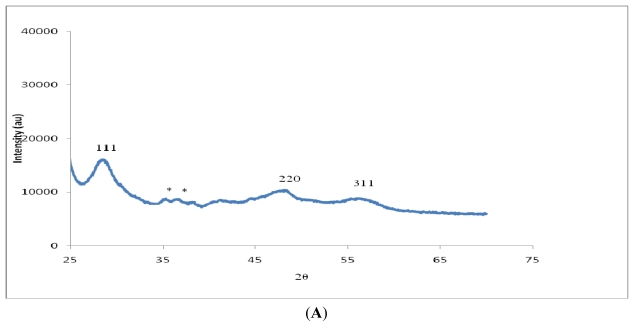

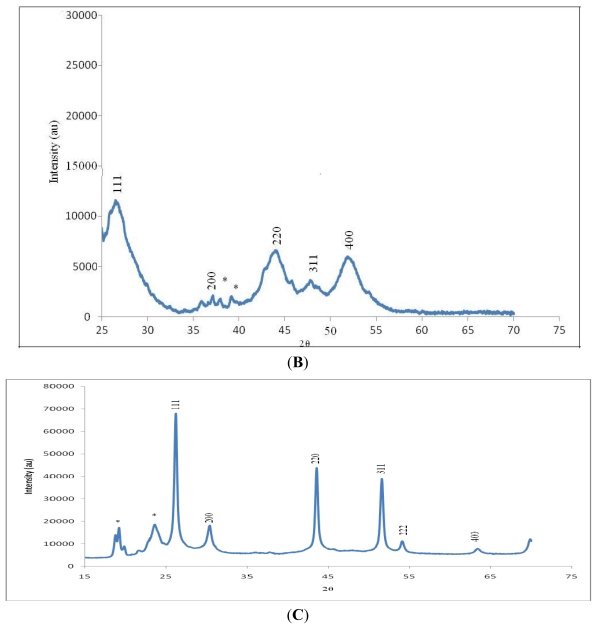
(**A**). Powder XRD pattern of ZnS nanoparticles; (**B**) Powder XRD pattern of CdS nanoparticles; (**C**) Powder XRD pattern of HgS nanoparticles.

The powder X-ray diffraction patterns for the nanoparticles are shown in Figures 3. The use of different alkyl groups on the same positions of the precursor complexes is likely to present nanoparticles of different properties owing to different mechanisms of formation since the substituents at the nitrogen of dithiocarbamate can influence the metal complex’s thermal decomposition [35]. ZnS display peaks (Figure 3A) which can be indexed to single phase sphalerite crystal structure (zinc blende) with lattice constant comparable to the values of JCPDS 05–0566. It is clear that the peaks are relatively broad, indicating that the nanoparticles have small size. The cadmium complex (Figure 3B) produced growth of particles in the hexagonal phase with XRD patterns indexed to 111, 200, 220, 311 and 400 which is in agreement with the reported value (JCPDS card, No. 5-0566). The peaks are broad, indicative of nanoscale particles. HgS exhibits diffraction peaks (Figure 3C) which correspond to the (111), (200), (220), (311), (222), and (400) planes of HgS (metacinnabar, syn), and are in good agreement with the pattern JCPDS 00-006-0261. The relative broadening of the peaks indicates that the particles are small in size. The diffraction peaks due to CdS and ZnS samples are relatively broader compared to those of HgS samples, indicating the formation of smaller CdS and ZnS nanoparticles than those of HgS.

### 2.4. Optical Characterization

#### 2.4.1. Absorption Studies

**Figure 4:**
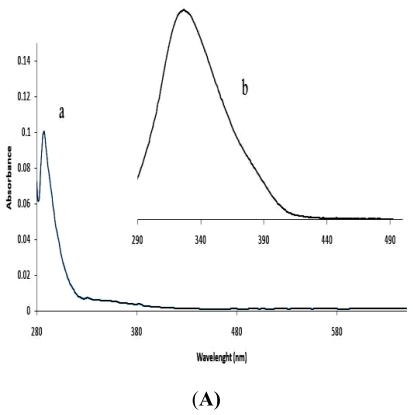

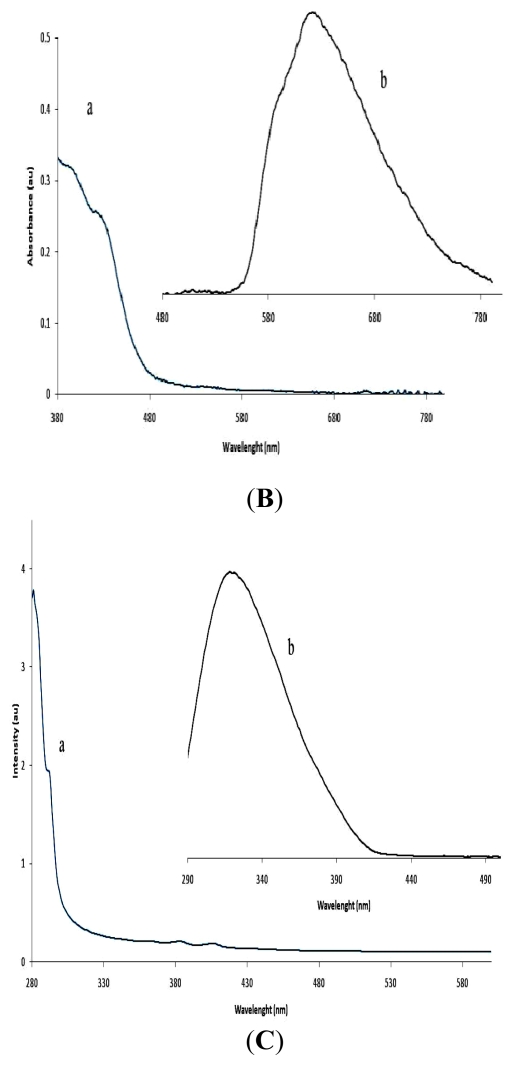
Absorption and emission spectra of the nanoparticles. (**A**) Absorption (a) and emission (b) spectra of ZnS nanoparticles; (**B**) Absorption (a) and emission (b) spectra of CdS nanoparticles; (**C**) Absorption (a) and emission (b) spectra of HgS nanoparticles.

The band gap energy in a nanomaterial could be obtained from the absorption maxima. According to quantum confinement theory, electrons in the conduction band and holes in the valence band are spatially confined by the potential barrier of the surface. Due to confinement of both electrons and holes, the lowest energy optical transition from the valence to conduction band will increase in energy, effectively increasing the bandgap (E_g_) [36]. The shoulder or peak of the spectra corresponds to the fundamental absorption edges in the samples, and could be used to estimate the band gap of the nanomaterial [37]. Measurements from the absorption peaks (Figure 4) gave the estimated band gap as 4.33 eV (286 nm), 2.91 eV (426 nm) and 4.27 eV (290 nm) for the ZnS, CdS and HgS samples respectively. The excitonic feature of the HgS sample is not very pronounced compared to ZnS and CdS samples. A blue shift of the absorption edge of the samples from their bulk value of 3.71 eV (334 nm) of ZnS [38], 2.40 eV (516 nm) of CdS [39], and 2.0 eV (620 nm) of HgS is clearly observed [40]. The blue shift in the band edge is a consequence of exciton confinement and confirms the presence of nanoparticulate materials.

According to Effective Mass Approximation, the band gap energy of a nanoparticle is related to its diameter [41,42] and is shown in the relationship, *E_g_^eff^* =*E_g_* +*ħ*^2^π^2^/2*μR*^2^. Where, *E_g_* is the bandgap of the bulk semiconductor, *h* is Planck’s constant, *R* is the radius of the nanoparticle, and *μ* is the reduced mass of the exciton given by *m*_e_ *m_h_*/(*m*_e_ + *m_h_*). Here, *m_e_* and *m_h_* are masses of the electron and hole, respectively. This model was expanded by Brus [43] to include Columbic interaction of excitons and the correlation energy and can be written as, $\Delta \;E_{g}=E_{g}^{nano}-E_{g}^{bulk}=\frac{h^{2}}{8r^{2}}\left(\frac{1}{m_{e}^{*}}+\frac{1}{m_{h}^{*}}\right)-\frac{1.8\;e^{2}}{4\;\pi ɛ{ɛ}_{o}\;r}$. Using this relation, the absorption edge is calculated from the intersection of the sharply decreasing region of the spectrum with the baseline [41]. We applied the Brus equation to the CdS and ZnS samples using the following parameters: ZnS (m_e_^*^ = 0.42*m*_0_, m_h_^*^ = 0.61*m*_0_) [44], CdS (m_e_^*^ = 0.21*m*_0_, m_h_^*^ = 0.80*m*_0_), where *m*_0_ is the mass of electron [45]. The estimated particles sizes are 4.82 and 3.54 nm for CdS and ZnS samples respectively. Since the effective mass of the electrons is much smaller than that of the holes, it implies that the charge carrier confinement mainly affects the energetic level of the electrons [46].

#### 2.4.2. Luminescence Studies

The pholuminescence (PL) spectra (Figure 4) of the as-synthesized nanoparticles were performed in order to investigate their luminescence properties. Two emission peaks have been observed for semiconductor nanocrystals and they are ascribed to the exciton and the trapped luminescence [47]. While the exciton emission peak appears as sharp band, the trapped emission is broad [48,49]. Only one emission band showed in the spectra of the as-synthesized nanoparticles. The three samples, ZnS, CdS and HgS, exhibit emission maximum at 328, 622 and 320 nm respectively. The strong band gap emission demonstrates the high crystalline nature of the as-synthesized particles. All the nanoparticles showed a red-shift with respect to the absorption edge. The observed broadening of the emission peak could be attributed to both the size distribution and the increase of the surface states due to the increase in surface to volume ratio for smaller nanoparticles [50].

## 3. Experimental Section

### 3.1. Materials

All the chemicals used, Hexadecylamine (HDA), trioctylphosphine (TOP), toluene and absolute methanol was purchased from Aldrich. They were all of analytical grade and were used without further purification. The precursor complexes were prepared and characterized as reported earlier [27]. The complexes are: (*N*,*N*–ethyl phenyl–N, N–butyl phenyl dithiocarbamato)Zn(II), [ZnL^1^L^2^]; (*N*,*N*–ethyl phenyl–*N*,*N*–butyl phenyl dithiocarbamato)Cd(II), [CdL^1^L^2^]; and (*N*,*N*–ethyl phenyl–*N*,*N*–butyl phenyl dithiocarbamato)Hg(II), [HgL^1^L^2^].

### 3.2. Preparation of Nanoparticles

Synthesis of the metal sulfide nanoparticles, chemically passivated with hexadecylamine was carried out as follows. In a typical experiment, [ZnL^1^L^2^] (0.7 g) was dissolved in TOP (15 mL). This solution was then injected into 7.5 g of hot HDA at a temperature of 180 °C. A subsequent decrease in temperature of 20–30 °C was observed. The solution was then allowed to attain 180 °C and then heated for a further 60 min. The solution was then allowed to cool to about 70 °C, and large amount of methanol was added to remove the excess HDA. The as-synthesised flocculent precipitate was separated by centrifugation and was redispersed in toluene. The solvent was then removed by evaporation under reduced pressure to give HDA-capped ZnS nanoparticles. The particles were again washed with methanol three times and redispersed in toluene for spectroscopic measurements. The same approach was used for [CdL^1^L^2^] and [HgL^1^L^2^] to prepare HDA-capped CdS and HgS nanoparticles respectively.

### 3.3. Sample Characterization

The optical measurements were carried out using Perkin Elmer Lambda 25 UV–Vis spectrophotometer at room temperature. The samples solution in toluene were placed in glass cuvettes (1 cm path length) using toluene as a reference solvent for the nanoparticles. A Perkin Elmer LS 45 Fluorimeter was used to measure the photoluminescence of the nanoparticles. Powder X-ray diffraction patterns were recorded on a Bruker D8, advanced equipped with a proportional counter, using Cu Kα radiation (λ = 1.5405 A, nickel filter). Measurements were taken at a high angle 2θ range of 20–90° using a scan speed of 0.01°, with a filter time-constant of 2.5 s per step and a slit width of 6.0 mm. The TEM images were obtained using a Philips CM 200 compustage electron microscope operated at 200 kV. The SEM images were obtained in a Jeol, JSM-6390 LV apparatus, using an accelerating voltage between 15–20 kV at different magnifications, as indicated on the SEM image. Composition and energy dispersive spectra were processed using energy dispersive X-ray analysis (EDX) attached to the SEM with Noran System Six software. The TGA was carried out using Perkin Elmer thermogravimetric analyzer (TGA 7) equipped with a thermal analysis controller (TAC 7/DX). The differential-scanning calorimetry at high temperature was performed with a Thermo scientific DSC (i–series) instrument.

## 4. Conclusions

Zn(II), Cd(II) and Hg(II) complexes of *N*,*N*’-ethyl phenyl-*N*,*N*’-butyl phenyl dithiocarbamate complexes was used as single source precursors for the preparation of metal sulfide nanoparticles. Thermogravimetric analysis showed that the complexes decomposed sharply to form their respective metal sulfides. Hexadecylamine capped ZnS, CdS, HgS nanoparticles were prepared by thermolyzing the complexes at 180 °C for 1 h. X-ray diffraction provides definite identification of the crystal structure and scanning electron and transmission electron micrographs confirmed the formation of different morphologies of the as-synthesized nanoparticles.

## Figures and Tables

**Table 1:** Summary of the thermal characteristics for the *N*,*N*-ethyl phenyl *N*,*N*-butyl phenyl dithiocarbamate complexes.

Compounds	Decompos. Ranges	Peak Temperature (°C)	Weight Loss (%)	Product Expected	Mass Changes	
					Calc.	Found
[Zn L^1^L^2^]	204–382	318	78.2	ZnS	2.32	2.85
[Cd L^1^L^2^]	229–372	323	70.0	CdS	2.85	3.10
[Hg L^1^L^2^]	205–340	302	78.92	HgS	4.34	4.60
